# Acknowledgement to Reviewers of *Toxins* in 2013

**DOI:** 10.3390/toxins6020779

**Published:** 2014-02-24

**Authors:** Toxins Editorial Office

**Affiliations:** MDPI AG, Klybeckstrasse 64, CH-4057 Basel, Switzerland; E-Mail: toxins@mdpi.com

The editors of *Toxins* would like to express their sincere gratitude to the following reviewers for assessing manuscripts in 2013: 

Abbas, HamedAyyalusamy, RamamoorthyBlahova, LucieAberg, Annica TevellBabica, PavelBlasi, JuanAbrunhosa, LuisBacker, Marina V.Blum, LoicAdam, GerhardBailly, Jean-DenisBokori-Brown, MonikaAdang, Michael J.Bammens, BertBooth, WarrenAdorini, LucianoBaptista, MafaldaBorchers, Christoph H.Adriano, MaroccoBarreau, ChristianBorucinska, Joanna D.Aktories, KlausBarth, HolgerBorutova, RadkaAlbrecht, Eric A.Basak, Ajit K.Bos, Mettine H.A.Ali, Syed AbidBastiaenen, Caroline H.G.Bosilevac, Joseph M.Altenmüller, EckhartBastos, MargaridaBosman, GielAmezqueta, SusanaBattilani, PaolaBosmans, FrankAmicarelli, FernandaBazin, IngridBouaïcha, NoureddineAmuzie, Chidozie J.Beck, JohannesBougis, PierreAnfossi, LauraBenoist, HervéBoukouvala, SotiriaApplegate, Todd J.Bentur, YedidiaBradley, Walter G.Arber, NadirBernhardt, IngolfBradshaw, RosieArgilés, ÀngelBerry, John P.Brasel, TrevorAriga, HiroyoshiBerthiller, FranzBraun, VolkmarAriño, AgustínBertuzzi, TerenzioBrautigan, David L.Armstrong, Glen D.Bidigare, Robert R.Brown, DarenArrigan, DamienBielaszewska, MartinaBruzzone, SantinaAsakawa, ManabuBierbaum, GabrieleBurke, ShannonAvanzini, GiulianoBilluart, PButtenschoen, KlausByrd, WyattDe Rijk, Theo C.Field, DesCai, ShuoweiDean, PaulFimoniari, CarmelaCaleo, MatteoDebreuil, DanielFiorentini, CarlaCalvete, Juan J.Deeds, Jonathan R.Fleming, ThomasCâmara, JoséDegen, Gisela H.Flower, DarrenCampos, AlexandreDegenkolb, ThomasFolinsbee, Kaila E.Camu, WilliamDel Pozo, Álvaro MartínezFrisan, TeresaCarmichael, WayneDell'Aversano, CarmelaFroelich, Christopher J.Cary, JeffreyDemeke, TigstFujii, ManabuCegielska-Radziejewska, RenataDeRosa, Maria C.Fujita, HiroshiCentonze, DiegoDesai, Kaushik M.Fuly, André LopesCerasino, LeonardoDevreese, MathiasGagliano, NicolettaCeravolo, Maria GabriellaDi Marco, PeterGalaverna, GianniChakravorty, AnjanaDi Pietro, NataliaGámiz-Gracia, LauraChang, Long-SenDias, ElsaGarb, Jessica E.Chang, XiaoqingDiaz-Orejas, RamonGarmendia, JunkalChatterjee, Som SubhraDickerson, Tobin J.Genuis, Stephen J.Cheli, FedericaDing, WeiGerwin, RobertChen, RongDinkova-Kostova, AlbenaGessani, SandraChiffoleau, EliseDobrindt, UlrichGhareeb, KhaledChitalia, VipulD'Ovidio, RenatoGibson, John S.Chock, Pweh BoonDowning, TimGlorieux, GrietChristensen, StephanieDoyle, SeanGohar, MichelChrpová, JanaDuarte, SofiaGoldstein, SteveCirés, SamuelDuca, RaduGong, Yun YunClarke, AnthonyDudziak, DianaGorman, AdrienneCohen, GeraldDufresne, MarieGrasso, LuigiCoronel, M.B.Duncan, Joseph A.Grauso, LauraCossart, PascaleDurek, ThomasGriffiths, Gareth D.Costa, JoãoDuvic, MadeleineGross, Giuseppe W.Cozzini, PietroEhrlich, KenGurevitz, MichaelCross, Alan S.Elliott, CandaceGutiérrez, SantiagoCroubels, SiskaEspiña, BegoñaGuzmán-Guillén, RemédiosCummings, Brian S.Esquenazi, A.Halter, DavidCunha, SaraFabbro, LarelleHamasaki, NaotakaCurtis, TheresaFactor-Litvak, PamHammond-Kosack, KimDaines, DayleFakhoury, AhmadHannestad, JonasDaniels-Wells, Tracy R.Falconer, Ian R.Hardy, Simon P.De Crécy-Lagard, ValérieFanelli, FrancescaHe, XiaohuaDe Girolamo, Annalisa Faure, GrazynaHégarat, LeDe Haro, LucFerreras, JosÉ MiguelHellmann, NadjaDe Kimpe, NorbertFessard, ValérieHendrich, SuzanneHenley, BillKonoki, KeiichiMadden, L.Herring, SusanKoppen, RobertMaghazachi, Azzam A.Hess, PhilippKornblau, Steven M.Manning, BruceHeuck, Alejandro P.Kovács, MelindaMantis, NicholasHill, DonnaKrienitz, LotharMantle, Peter G.Hirai, HirofumiKrnjaja, VesnaMarchesini, Gerardo R.Hodgson, Wayne C.Krock, BerndMaresca, MarcHofmann, Alan F.Kubicek, Christian PMarie, BenjaminHoole, DaveKurmayer, RainerMariottini, Gian LuigiHuertas-Pérez, José F.Kushiro, MasayoMarkaki, PanagiotaHungerford, Dr JamesKusnecov, Alexander W.Markland, FrankIannitti, TommasoKuzdraliński, AdamMarrs, CarlIliev, AsparouhLacadena, JavierMartin-Eauclaire, Marie-FranceInagaki, HidetoshiLacaze, Jean PierreMartner, AnnaIsbister, Geoffrey K.Lacy, D. BordenMaruyama, ToruIshii, TetsuroLagarde, FlorenceMarzocco, StefaniaJaen, Maria TeresaLangenhan, TobiasMascini, MacelloJorgensen, ReneLannin, NatashaMason, KevinJoshi, BharatLaPlace-Treyture, ChristopheMatsuda, ShigeakiJungblut, Peter R.Lattanzio, VeronicaMatsuda, YasunobuKaempfer, RaymondLazaridis, ThemisMaul, RonaldKahlert, StefanLe Bourhis, Xuefen McArdle, Joseph J.Kakinuma, YoshihikoLedizet, MichelMccabe, Paul F.Kamitani, ShigekiLee, Maw-RongMcgee, David J.Kamouchi, MasahiroLehotay, Steven J.Mcgregor, Glenn B.Kang, ChulHeeLeipold, EnricoMckillip, John L.Karimi, KhalilLemichez, EmmanuelMearini, LuigiKarp, Barbara IllowskyLemmens-Gruber, RosaMeca, GiuseppeKatikou, PanagiotaLeung, NelsonMedeiros, Bruno C.Kazan, KemalLiagre, BertrandMeijers, BjörnKhanna, RajeshLimón, M. CarmenMekada, EisukeKim, BumseokLin, HeningMelloni, EdonKim, H.Lin, Hsi-hsienMelo, M.M.Kim, Ji HoeLindbäck, TorilMelton-Celsa, AngelaKim, Kwang SikLisacek, FrederiqueMerel, SylvainKim, Sung OukLiu, Biing-HuiMerrill, A. RodKintzios, SpiridonLoboda, A.Metcalf, JamesKishida, TakushiLópez de Cerain, Adela LópezMijakovic, IvanKnapp, OliverLord, MikeMiller, DavidKoch , Matthias Lozovaya, Vera V.Minervini, FiorenzaKomericki, PeterLuongo, DiomiraMiranda, DanteKong, Siu-KaiMa, DongyingMizuno, MasashiMohanraj, RajeshPark, Pyong WooRediske, Richard R.Mollica, AdrianoPascale, MichelangeloReverberi, MassimoMonaci, LindaPassi, AlbertoRhodes, JonathanMoran, YehuPattono, DanieleRicci, VittorioMoreno, Isabel MaríaPeigneur, SteveRicciardi, LucianaMoretti, AntonioPellett, SabineRichard-Forget, FlorenceMorita, KyojiPerrone, JensRigosi, AnnaMourao, Paulo AntonioPeter, KarlheinzRimbach, GeraldMoxley, Rodney A.Peterson, Stephen W.Rodrigues, InêsMukherjea, DebashreePfohl-Leszkowicz, AnnieRodriguez de la Vega, RicardoMukherjee, SumanPhillips, T. D.Rounge, Trine B.Müller, UlrichPierce, Samuel R.Rubert, J.Munday, RexPincus, SethRubio, VicenteMurch, SusanPinton, PhilippeRuiz, María-JoséMüthing, JohannesPizzo, Salvatore V.Russo, RayNagahama, MasahiroPletinck, AnneleenSabart, MarionNagata, KojiPlomp, JaapSainis, IoannisNakakita, Shin-ichiPohl, Mary AnnSaito, HideyukiNakane, AkioPolak-Jonkisz, DorotaSaito, YoshiroNakano, TakanariPoli, MarkSalmaso, NicoNashan, BjörnPolito, LetiziaSalter, RussellNeffling, Milla-riinaPolo-Parada, LuisSánchez, Elda E.Nemunaitis, JohnPonce-Soto, Luis AlbertoSantamato, AndreaNeri, CristinaPoniedziałek, BarbaraSantos, Regiane R.Neuberg, DonnaPopoff, Michel R.Sarkar, Nurul H.Niewold, TheoPosthuma, TruusSauer, John-DemianNishikawa, YoshikazuPraetorius, Helle A.Sayner, Sarah L.Noguchi, TamaoPrelle, AmbraSchaafsma, ArtO’callaghan, Christopher A.Prosperini, AlessandraSchäfer, WilhelmOikawa, HideakiPurohit, PrasadSchaffner, DonaldOkumura, TadayoshiPuschner, BirgitSchardl, Christopher L.Opitz, BastianPusztahelyi, TündeScharf, Daniel H.Orth, Joachim H. C.Rahman, M. SayeedurScheffler, KonradOsbourn, AnneRajagopalan, GovindSchlapbach, ChristophOtten, TimothyRanganathan, NatarajanSchmidt, GudulaOwens, RoisinRaphael, BrianSchmidt, H.Pagan, Fernando L.Rasmussen, Peter HaveSchuller, Hildegard M.Palermo, Marina S.Rasooly, ReuvenSchwartz, Elisabeth FPalm-Apergi, CarolineRatner, Adam J.Scollenberger, MargitPaoli, MarcoRay, Rumiana V.Scott, BarryParent-Massin, DominiqueReason, DonaldSeitz, MarenPark, JooyounRecio-Pinto, EsperanzaSepulveda, RosarioSerrano, Ana BelénSwevers, LucWarth, BenediktSerrano, RoqueTadahiro, SuzukiWarzecha, TomaszSeth, RohitTakagi, MasahiroWeller, Michael G.Seveau, StephanieTan, Eng-KingWiegand, ClaudiaShao, DongminTanida, SatoshiWiemann, PhilippSieroslawska, AnnaTatulian, SurenWilliams, David E.Słomińska-Wojewódzka, MonikaTesh, VernonWilliams, Gary M.Smith, Laura E.Tetreau, GuillaumeWilson, BrendaSolana, RafaelThakur, MayankWilson, PaulSolfrizzo, M.Tolleson, William H.Witzenrath, MartinSong, SuquanTorres, VictorWladyka, BenedyktSørensen, Jens LauridsTortorella, DomenicoWood, Susie A.Spink, David C.Tsushima, SeiyaWood, Thomas K.Spoof, LisaTubaro, AureliaWright, Christine E.Spooner, RobertTudzynski, BettinaWu, Alan H.B.Stamelou, MariaUgalde, UnaiWu, Chih-JenStamopoulos, DimosthenisUhlig, SilvioWu, YuliangStępień, ŁukaszUrquhart, Bradley L.Würzner, ReinhardStewart, Linda D.Valenti, GiovanniWycoff, Keith L.Stiles, Bradvan Bruggen, RobinWyllie, David H.Stiles, Bradley G.Van Den Berg, Marco AlexanderYakes, BetsyStirpe, FiorenzoVan Wichelen, JeroenYamamoto, AtsushiStokes, Amber N.Varga, JánosYamamoto, YoshimasaStournaras, ChristosVarnum, SusanYoon, Moon-YoungStrasser, AloisVasconcelos, VitorYoshinari, TomoyaStrotman, LindsayVerbrugghe, ElinYu, JiujiangStruck, Manuel FlorianVetter, IrinaZamarin, DmitriySukharev, SergeiVettorazzi, A.Zeng, YanSulyok, MichaelVonk, Freek J.Ziková, AndreaSuman, MicheleVoss, KennethZobel-Thropp, Pamela A.Surai, PeterWälchli, SébastienZoja, CarlaSuzuki, HodakaWang, JunZöllner, PeterSuzuki-Inoue, KatsueWarrell, David A.

